# Antifungal activity of water-stable copper-containing metal-organic frameworks

**DOI:** 10.1098/rsos.170654

**Published:** 2017-10-11

**Authors:** Supaporn Bouson, Atiweena Krittayavathananon, Nutthaphon Phattharasupakun, Patcharaporn Siwayaprahm, Montree Sawangphruk

**Affiliations:** 1 Department of Microbiology, Faculty of Science, Kasetsart University, Bangkok 10900, Thailand; 2 Department of Chemical and Biomolecular Engineering, School of Energy Science and Technology, Vidyasirimedhi Institute of Science and Technology, Rayong 21210, Thailand

**Keywords:** copper-based benzenetricarboxylate, metal-organic frameworks, porous coordination polymers, antifungal, antimicrobial

## Abstract

Although metal-organic frameworks (MOFs) or porous coordination polymers have been widely studied, their antimicrobial activities have not yet been fully investigated. In this work, antifungal activity of copper-based benzene-tricarboxylate MOF (Cu-BTC MOF), which is water stable and industrially interesting, is investigated against *Candida albicans*, *Aspergillus niger*, *Aspergillus oryzae* and *Fusarium oxysporum*. The Cu-BTC MOF can effectively inhibit the growth rate of *C. albicans* and remarkably inhibit the spore growth of *A. niger*, *A. oryzae* and *F. oxysporum*. This finding shows the potential of using Cu-BTC MOF as a strong biocidal material against representative yeasts and moulds that are commonly found in the food and agricultural industries.

## 1. Introduction

Metal-organic frameworks (MOFs) or porous coordination polymers are a group of highly porous crystalline materials consisting of metal clusters interconnected with organic linkers [1–6]. Owing to their high surface area and other tunable properties, they have been extensively investigating in many applications since their discovery [1,5,6]. Copper-1,3,5-benzenetricarboxylate (Cu-BTC), which is water stable [7], is one of the most recognized MOFs widely investigated for many applications. A Cu^2+^ dimer ion is located at the centre of the structure where each copper atom is connected by four oxygen atoms from the benzenetricarboxylate (BTC) linker and one water molecule, with a general formula of Cu_3_(BTC)_2_(H_2_O)_3_. A binuclear Cu_2_ paddle wheel unit is connected into a three-dimensional structure of an octahedron shape with square-shaped pores (9 Å by 9 Å) [8]. Cu-BTC is used in many applications such as sensors [9], gas absorption [10] and membrane-based separation [11] because it has high surface area (600–1600 m^2^ g^−1^), large pore volume (approx. 0.7 cm^3^ g^−1^) and good thermal stability (up to 350°C) [12]. While Cu-BTC is being used in practical applications, its antifungal property, which is very important for environmental reasons and for animals including humans, has not yet been fully investigated. Also, it was reported that Cu-BTC can reduce oxygen gas producing the scavenger reactive oxygen species (ROS), which can inhibit microorganisms.

It is necessary to note here that Cu ions have a powerful antibacterial activity on both Gram-negative and Gram-positive bacteria as well as antifungal activity [13,14]. The reduction of Cu (II) ions to Cu (I) ions can generate superoxide species resulting in the degradation of biomolecules [15]. Some previous reports showed that metal ions can be derived from the culture medium, interrupting the ATP production process and disrupting DNA replication [16]. However, Cu-BTC and other Cu-containing MOFs are water stable [7] so it is interesting to investigate their antimicrobial activities in the culture environment.

Fungi are a member of eukaryotic organisms including yeasts and moulds. Among various resourceful fungal pathogens, *Candida* sp., *Aspergillus* sp. and *Fusarium* sp. cause serious and common infections. *Candida albicans* is a dimorphic fungus that can form both yeast and filamentous cells, typically contaminating foods leading to foodborne disease and food spoilage. In some certain clinical situations, *C. albicans* may become virulent [17] because it can disseminate through the bloodstream. Note, the contamination of foodborne pathogens and food spoilage microorganisms is one of the most concerning issues in the food industry. In addition, another cause of food spoilage comes from *Aspergillus* species. *Aspergillus niger* and *A. oryzae* are usually found in bakery products, intermediate-moisture food products, cheeses, preserved fruits and grains. As a result, *C. albicans, A. niger* and *A. oryzae* were selected as the infectious fungi to be tested with the Cu-BTC. In addition, a fungus namely *Fusarium oxysporum*, typically found in soils leading to common adulteration in agricultural products, was also chosen as a representative of fungi because it has a huge impact on the environment. This fungus can grow on organic matter in soils and the rhizosphere in plants, which can give rise to saprophytic plant pathogens [18].

Although there is no previous report on the antifungal activity of Cu-BTC against *C. albicans, A. niger*, *A. oryzae* and *F. oxysporum*, HKUSR-1 MOF can inhibit *Saccharomyces cerevisiae* [19]. Our results here interestingly show that the Cu-BTC, which is water stable, can strongly inhibit the growth of *C. albicans*, *A. niger*, *A. oryzae* and *F. oxysporum* in the culture media.

## 2. Material and methods

### 2.1. Chemicals and materials

Copper nitrate trihydrate (99.5%, Lobal Chemie), trimesic acid (95%, Sigma-Aldrich), *N*,*N*-dimethylformamide (DMF; 99.8%, QRec), ethanol (99.9%, QRec), Sabouraud Dextrose Agar (SDA; Himedia), Sabouraud Dextrose Broth (SDB; Himedia) and Potato Dextrose Agar (PDA; Himedia) were of analytical reagent grade. All chemical compounds were diluted with deionized water (greater than 18 MΩ cm, Millipore).

### 2.2. Synthesis of copper-based benzenetricarboxylate

Cu-BTC (HKUST-1) was synthesized by a conventional hydrothermal method previously reported [20,21]. Briefly, 12-mM Cu(NO_3_)_2_·3H_2_O was dissolved in deionized water (25 ml) and stirred for 15 min. Trimesic acid ligand (8 mM) was dissolved in DMF (25 ml). The two solutions were then mixed together by stirring at room temperature (25°C) for 10 min. The mixture was transferred to a Teflon-lined stainless-steel autoclave and heated at 110°C for 24 h. After the reaction, it was cooled naturally to room temperature. Blue crystals of Cu-BTC were recovered by washing with ethanol and deionized water for five times and dried in a vacuum oven at 100°C for 24 h. The as-synthesized material was characterized by field-emission scanning electron microscopy (FE-SEM; Philips: XL30), transmission electron microscopy (TEM; EI Tecnai G2, Eindhoven, The Netherlands), Fourier transform infrared (FTIR; Perkin Elmer System 2000) and X-ray diffraction (XRD; Philips X'Pert). To confirm that Cu-BTC is water stable, 2 g Cu-BTC was immersed in 50 ml deionized water for a week and filtered by a vacuum filtration. The element in the filtered water was measured by wavelength-dispersive X-ray fluorescent spectroscopy (WDXRF; Bruker, S8 Tiger model, Germany). The details of WDXRF measurement can be found in the electronic supplementary material.

### 2.3. Antifungal assays for yeasts and moulds

*Candida albicans* (TISTR 5779), a yeast, was obtained from a culture collection at Thailand Institute of Scientific and Technological Research. The assay was prepared by cultivating the microorganism in SDB medium under shaking at 150 r.p.m. at 25°C for 16–18 h. The culture was sterile diluted in a saline solution to reach a concentration of approximately 1.5 × 10^8^ colony forming units (CFU) per millilitre. For the fungicidal rate test, the Cu-BTC suspension (100–500 ppm) was mixed with the fungal suspension at a volume ratio of 1 : 2. The culture suspension was shaken at 150 r.p.m. at 25°C for 15, 30, 45 and 60 min, respectively after which 100 µl of culture suspension was spread on SDA plates. The number of viable fungal colonies was counted after incubation for 48 h at 25°C. Note, to confirm that Cu-BTC is stable in the culture, the culture with 500 ppm Cu-BTC was washed by vacuum filtration five times. The element in the filtered water was measured by WDXRF.

At the same time, three moulds including *A. niger*, *A. oryzae* and *F. oxysporum* were also selected to test the antifungal activity of Cu-BTC. The antifungal activity assay was determined by a poison food technique because those fungi are normally found contaminating foods [22]. Briefly, young active mycelial colonies of fungi were prepared by inoculating all fungi on PDA for 7 days at 25°C. The Cu-BTC was then added into each flask of PDA at different concentrations (100–500 ppm) and the suspension was shaken gently. The mixed suspension of PDA and Cu-BTC was then poured onto sterile Petri plates and inoculated with fungal plugs (8 mm in diameter). The inoculation was at 25°C for 7–10 days. Each condition was performed in triplicate wells. The diameter of mycelial growth in each Petri dish was measured and recorded. The PDA without Cu-BTC was also used as a negative control. The antifungal activity of each sample was evaluated as the percentage inhibition of mycelium growth as follows:

2.1 $$\%\;\text{inhibition of the mycelial growth}=\frac{\left(d_{c}-d_{t}\right)}{d_{c}}\times 100,$$

where *d*_c_ is the colony diameter (millimetres) of the control; *d*_t_ is the colony diameter (millimetres) of the treated disc.

The percentage inhibition was reported based on an average value of three replicates.

### 2.4. Morphologies of microorganisms with and without copper-based benzenetricarboxylate treatments

For yeast, *C. albicans* was cultivated in SDB medium under shaking at 150 r.p.m. at 25°C for 16–18 h. The culture was sterile diluted in a saline solution to a concentration of approximately 1.5 × 10^8^ CFU per millilitre. The culture suspension was mixed and inoculated with Cu-BTC at a concentration of 500 ppm under shaking at 150 r.p.m. at 25°C for 60 min. Next, the suspension was filtered and dehydrated with different concentrations of ethanol (50%, 70%, 80%, 90%, 95% and 100%, respectively) for 15–20 min at each step and put into a critical point dryer using liquid CO_2_. Finally, the dehydrated samples were coated with gold–palladium for SEM analysis (Quanta 450, The Netherlands).

For moulds, the Petri plates with an 8 mm diameter mycelium at the centre of the disc were inoculated with PDA mixed with Cu-BTC at different concentrations (100–500 ppm) at 25°C for 7 days. After inoculation, the peripheral mycelial disc was taken for SEM analysis. The samples were dehydrated with different concentrations of ethanol (50%, 70%, 80%, 90%, 95% and 100%, respectively) for 15–20 min at each step and put into a critical point dryer using liquid CO_2_. Finally, the dehydrated samples were coated with gold–palladium for SEM analysis.

## 3. Results and discussion

### 3.1. Morphological and structural properties of copper-based benzene tricarboxylate

**Figure 1.**
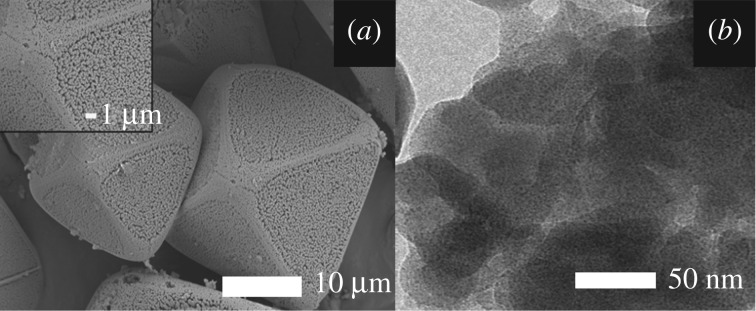
(*a*) FE-SEM and (*b*) TEM images of Cu-BTC.

After 2 g Cu-BTC was immersed in 50 ml deionized water for a week and then filtered by vacuum filtration, the WDXRF of the filtered water shows no copper indicating that the Cu-BTC is water stable. The morphology of the as-synthesized Cu-BTC was investigated by FE-SEM and TEM as shown in figure 1. The microstructure (10–20 µm in size) with an octahedral morphology of Cu-BTC is similar to another previous report [20]. It has a sponge-like structure containing a rough surface with uniform pores.

**Figure 2.**
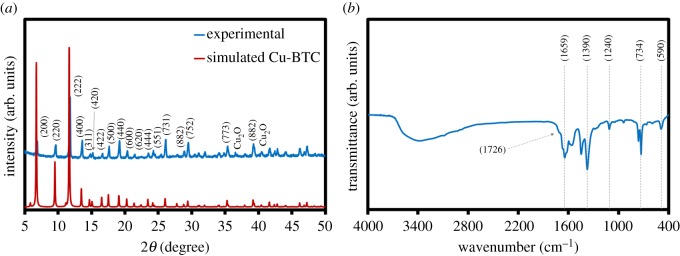
(*a*) XRD and (*b*) FTIR of Cu-BTC.

X-ray diffraction pattern (XRD) analysis was carried out to study the crystalline structure of the as-synthesized material as shown in figure 2*a*. All observed major peaks at 9.5° (220), 11.7° (222), 13.5° (400), 14.7° (331), 16.5° (422), 17.5° (511), 19.1° (440) and other minor peaks are the characteristics of pure Cu-BTC with a face centre cubic (FCC) structure, which are in good agreement with other previous work [20,23,24].

The FTIR spectrum (figure 2*b*) also confirms the characteristics of Cu-BTC. Two peaks observed at 590 and 734 cm^−1^ are attributed to the vibrational modes of Cu-O. The peaks between 1390 and 1659 cm^−1^ represent the stretching modes of the BTC ligand [25,26]. A small peak around 1726 cm^−1^ can be assigned to the vibrational mode of the carboxylic group in trimesic acid [27]. This carboxylate ion (COO^−^) is coordinated with two Cu(II) ions by bridging via a syn‒syn configuration [28]. The peaks around 1240 and 1659 cm^−1^ relate to the epoxy/peroxide and oxygen groups [29,30]. The broad peak around 3500 cm^−1^ is due to the coordinated water.

### 3.2. Antifungal activity of copper-based benzenetricarboxylate against yeast

**Figure 3.**
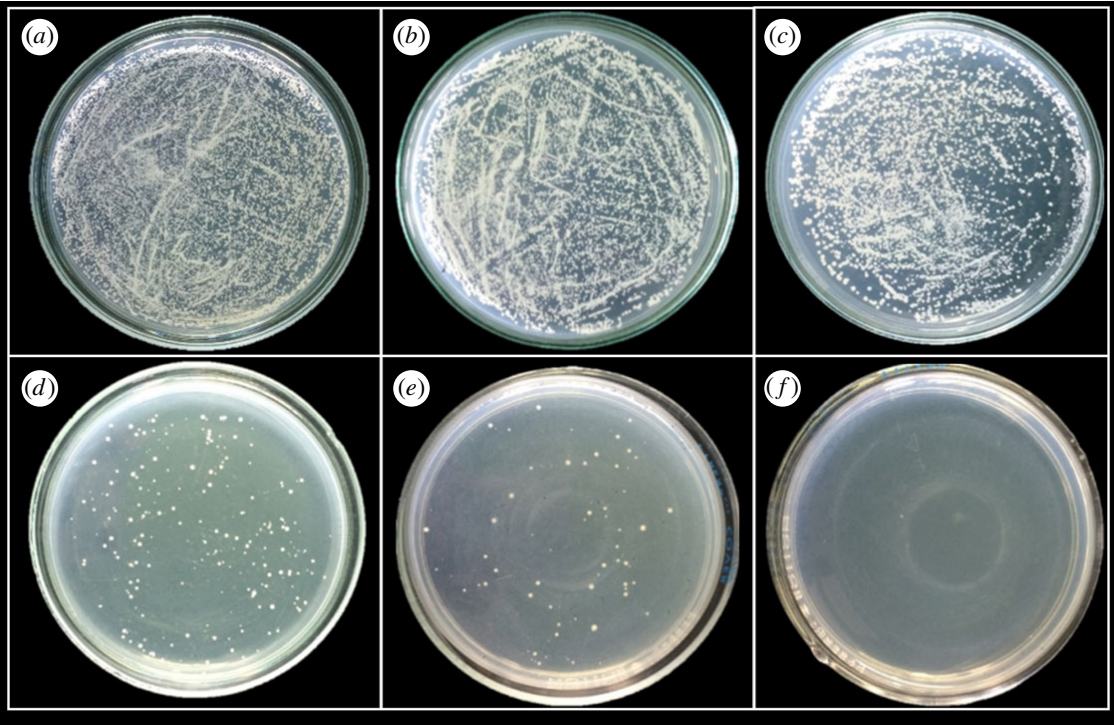
Antifungal properties of Cu-BTC at different concentrations against *C. albicans* after incubation for 60 min: (*a*) control, (*b*) 100 ppm, (*c*) 200 ppm, (*d*) 300 ppm, (*e*) 400 ppm and (*f*) 500 ppm.

To study the antifungal properties of Cu-BTC against *C. albicans*, which is a representative of yeasts, the dilution plate count technique was used. Different concentrations of Cu-BTC (100, 200, 300, 400 and 500 ppm) were incubated for 60 min and spread on SDA plates as shown in figure 3. It was found that the colonies are significantly reduced when increasing the concentrations of Cu-BTC. The Cu-BTC can inhibit *C. albicans* by approximately 96% at 300 ppm and up to 100% at 500 ppm.

In addition, the incubation time is another important factor for inhibiting the growth of *C. albicans*. The number of *C. albicans* colonies after treatment with Cu-BTC at different concentrations and incubation time were counted at 25°C. It was found that 500 ppm of Cu-BTC and the incubation time of 60 min are the most effective conditions for inhibiting the growth of *C. albicans* when compared with other conditions and other previously related work [19]. It can be concluded here that *C. albicans*, which is a dimorphic fungus, e.g. yeast and filamentous cell [31], can be inhibited by Cu-BTC.

**Figure 4.**
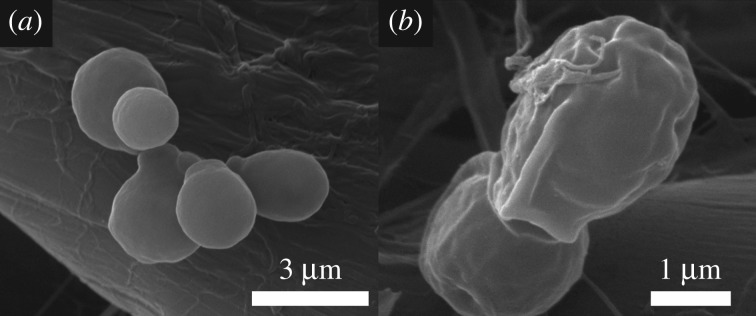
SEM images of *C. albicans* (*a*) without Cu-BTC treatment and (*b*) after treatment with Cu-BTC.

To further confirm the result of the antifungal assay, the morphology of *C. albicans* after treatment with Cu-BTC was compared with the control sample, the untreated *C. albicans*. The control sample is shown in figure 4*a* to present a smooth surface of *C. albicans*, as seen in another previous report [32]. After treatment with 500 ppm of Cu-BTC at 60 min (figure 4*b*), *C. albicans* has many wrinkles with a rough surface. This can be attributed to the damage of the cell membrane thanks to the leakage of intracellular compounds leading to complete cell disruption. This result is rather similar to other previous reports using other antimicrobial nanomaterials such as ZnO nanoparticles, Ag nanoparticles, Ag–ZnO nanocomposites and reduced graphene oxide [32–34], indicating the powerful antifungal activity of Cu-BTC against yeast. This is most probably due to the fact that Cu-BTC can reduce oxygen gas, producing the ROS, which can eventually inhibit the microorganisms. Note, the Cu-BTC is stable in the culture because the WDXRF of the filtered water washed from the culture with 500 ppm Cu-BTC shows no copper.

### 3.3. Antifungal activity of copper-based benzenetricarboxylate against moulds

**Figure 5.**
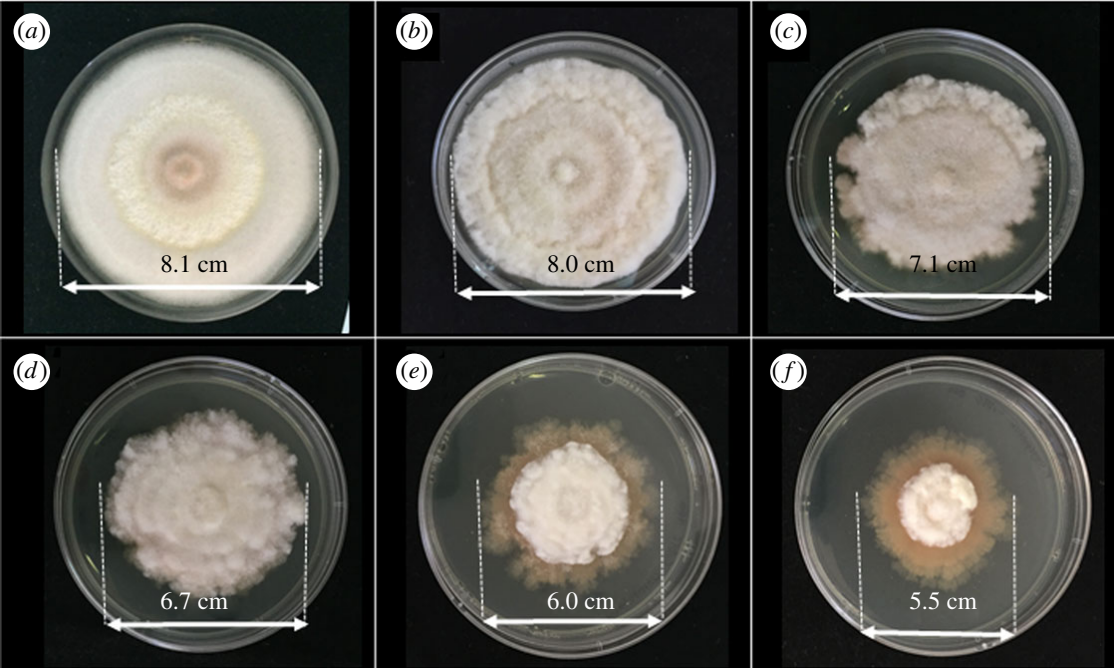
Photographs showing the mycelial growth of *F. oxysporum* on PDA medium containing different concentrations of Cu-BTC: (*a*) control or 0 ppm, (*b*) 100 ppm, (*c*) 200 ppm, (*d*) 300 ppm, (*e*) 400 ppm and (*f*) 500 ppm.

**Figure 6.**
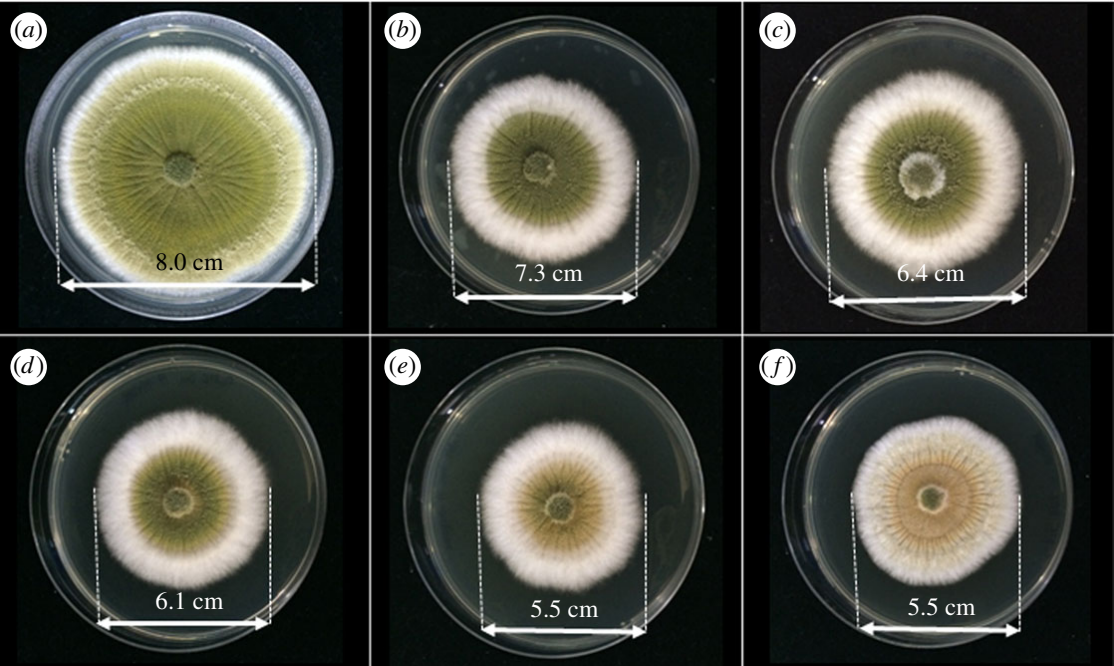
Photographs showing the mycelial growth of *A. oryzae* on PDA medium containing different concentrations of Cu-BTC: (*a*) control or 0 ppm, (*b*) 100 ppm, (*c*) 200 ppm, (*d*) 300 ppm, (*e*) 400 ppm and (*f*) 500 ppm.

**Figure 7.**
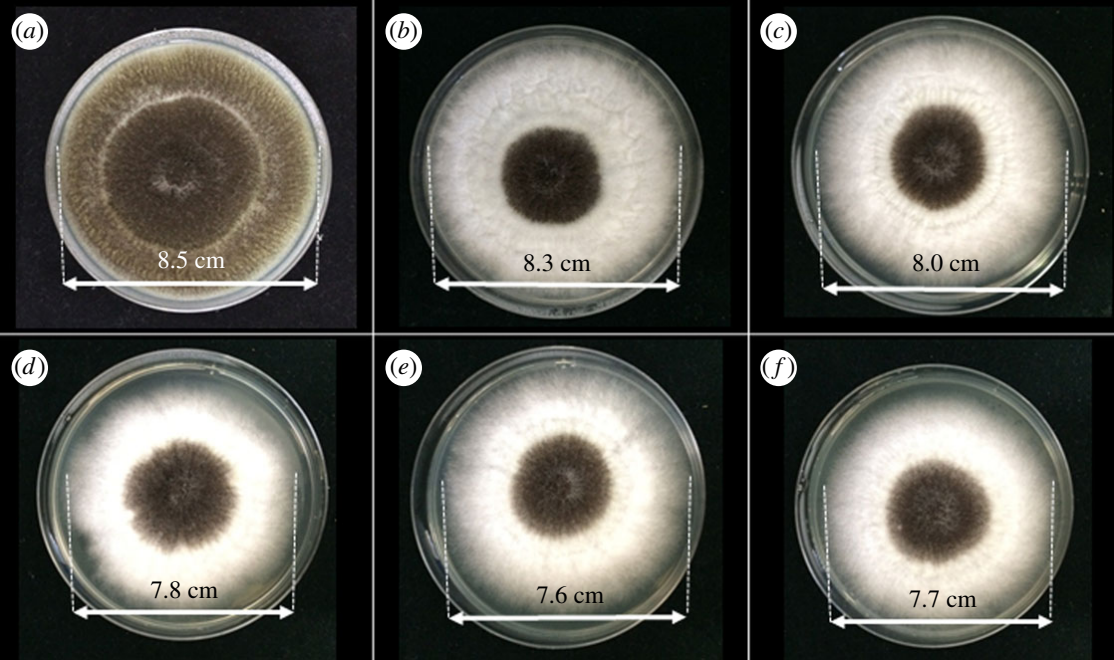
Photographs showing the mycelial growth of *A. niger* on PDA medium containing different concentrations of Cu-BTC: (*a*) control or 0 ppm, (*b*) 100 ppm, (*c*) 200 ppm, (*d*) 300 ppm, (*e*) 400 ppm and (*f*) 500 ppm.

To investigate the antifungal activity of Cu-BTC on moulds, the poison food technique or assay was used. In this experiment, three species of fungi (i.e. *F. oxysporum*, *A. oryzae* and *A. niger*), which are normally found in contaminated foods, were selected as the representatives of the moulds [18]. The results of the fungi with and without treatment with Cu-BTC are shown in figures 5–7. The effect of Cu-BTC on the mycelial growth of the three fungi is clearly observed, with smaller diameters of mycelial growth when treated with Cu-BTC. For *F. oxysporum* (figure 5) and *A. oryzae* (figure 6), the extent of mycelial growth inhibition significantly depends on the concentration of Cu-BTC. As the concentration of Cu-BTC increases, the inhibition rate also increases. At the highest concentration (500 ppm) of Cu-BTC, the percentage inhibition of Cu-BTC against *F. oxysporum* and *A. oryzae* is approximately 30*%*, as shown in table 1. However, after *A. niger* was incubated for 7 days on the Cu-BTC-containing PDA, there is no significant effect on the *A. niger* growth. This is in accord with another previous report using copper (C11000) and aluminium to inhibit *F. oxysporum* but not *A. niger* [35].

**Table 1.** Inhibition (%) of the mycelial growth of *F*. *oxysporum, A*. *oryzae* and *A*. *niger.*

	inhibition (%) of the mycelial growth				
fungi	100 ppm	200 ppm	300 ppm	400 ppm	500 ppm
*F. oxysporum*	1.23	12.35	17.28	25.93	32.10
*A. oryzae*	8.75	20.00	23.75	31.25	31.25
*A. niger*	2.35	5.88	8.24	10.59	9.41

Although the Cu-BTC cannot completely inhibit the fungi, it can remarkably affect the spore growth. This can be observed by the change in the spore colour of *A****.*** *oryzae,* which turns from green to pale yellow (figure 6). The change in colour can be explained by the inhibitory effect on the growth of spores***.*** In addition, the diameter of spore growth on these fungi is significantly reduced over 60%, resulting from the toxicity of distributed copper ions in the culture medium. The Cu-BTC may generate ROS that can interact with biomolecules in the fungal spores, inhibiting the fungi [36].

**Figure 8.**
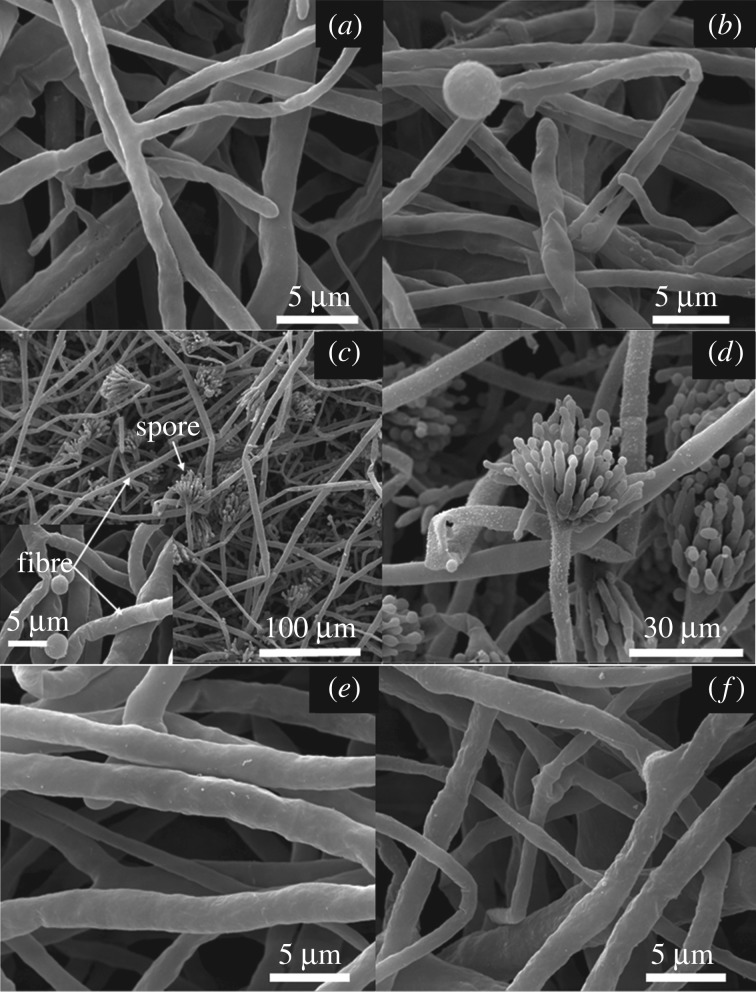
SEM images of *F. oxysporum*, (*a*) without Cu-BTC and (*b*) after treatment with Cu-BTC; SEM images of *A. oryzae*, (*c*) without Cu-BTC and (*d*) after treatment with Cu-BTC; and SEM images of *A. niger*, (*e*) without Cu-BTC and (*f*) after treatment with Cu-BTC.

To further confirm the antifungal activities of Cu-BTC against the mycelial growth of *F. oxysporum, A. oryzae* and *A. niger*, the SEM images shown in figure 8 reveal the effect of Cu-BTC treatment on the fungal morphologies. The mycelial morphology (figure 8*b*) of *F. oxysporum* after treatment with Cu-BTC was shrunk when compared with the untreated morphology (figure 8*a*). For *Aspergillus* species including *A. Oryzae* (figure 8*c*) and *A. niger* (figure 8*e*), the hyphae surface after treatment with Cu-BTC becomes rough with granules on the surfaces of *A. Oryzae* (figure 8*d*) and *A. niger* (figure 8*f*), respectively. Note, the result here is quite similar to the inhibition of *Trichophyton rubrum* by allicin and garlic extracts previously reported [37], where its mycelial. Its mycelium growth was decreased after treatment. However, the clear inhibitory mechanism of these fungi needs further studies.

## 4. Conclusion

Cu-BTC, which is water stable, shows a strong inhibitory activity against *C. albicans*, *A*. *niger*, *A*. *oryzae* and *F. oxysporum*. The Cu-BTC can damage the cell membrane resulting in the leakage of intracellular compounds leading to complete cell disruption and eventually death. As the Cu-BTC concentration increases, the inhibitory efficiency also increases. An amount of 500 ppm Cu-BTC and an incubation time of 60 min are found to be the most effective conditions for inhibiting the growth of *C. albicans.* For *A*. *niger*, *A*. *oryzae* and *F. oxysporum,* the Cu-BTC does inhibit the growth process of spores. The antifungal mechanism of Cu-BTC is most possibly based on the ROS generated via the oxygen reduction of oxygen gas by the Cu-BTC. The finding in this work may lead a new antifungal application of Cu-BTC. By contrast, it may lead to awareness of using Cu-BTC, which may have a negative impact on useful microorganisms in the environment. Also, further investigation on the antifungal mechanism of Cu-BTC and other water stable MOFs is needed.

## Supplementary Material

Wavelength Dispersive X-ray Fluorescence (WDXRF)

## Data accessibility

The details of WDXRF measurement can be found in the electronic supplementary material.
