# Supplementary material for: Antifungal activity of water-stable copper-containing metal-organic frameworks
Source: R Soc Open Sci. 2017 Oct 11;4(10):170654. doi: 10.1098/rsos.170654 (PMC5666258; doi:10.1098/rsos.170654)
Supplement: Wavelength Dispersive X-ray Fluorescence (WDXRF) [file rsos170654supp1.docx]

**Supporting Inforamtion**

Antifungal activity of water-stable copper-containing metal-organic frameworks

Supaporn Bouson,^a^ Atiweena Krittayavathananon,^b^ Nutthaphon Phattharasupakun,^b^ Patcharaporn Siwayaprahm^a*^ and Montree Sawangphruk^b*^

^a^Department of Microbiology, Faculty of Science, Kasetsart University, Bangkok 10900, Thailand * [fscippsp@ku.ac.th](mailto:fscippsp@ku.ac.th)

^b^Department of Chemical and Biomolecular Engineering, School of Energy Science and Technology, Vidyasirimedhi Institute of Science and Technology, Rayong 21210, Thailand * [montree.s@vistec.ac.th](mailto:montree.s@vistec.ac.th)

**WDXRF measurement**

The flowchart of WDXRF sample preparation is shown in Figure S1. First, a cup was prepared by inserting the prolene film (a thickness of 4.0 µm, Chemplex industries, inc.) between an outer sleeve and an inner sleeve by placing the inner sleeve with the larger end downward and placing the outer sleeve on top (Figure S1b). Then, the sample (culture medium with and without copper) in a petri dish (ca. 16.5 g) was transferred into the WDXRF sample cup inside the stainless steel holder (a diameter of 34 mm) (Figure S1d). The cap was then placed on top of the cup (Figure S1e) and the holder was then taken to the WDXRF chamber (Figure S1f) to perform the measurement. In case of the filtered water from culture medium with and without copper, the culture medium (2.0 g) was filtered with 50 ml DI water. After that, 25 ml of the filtered water was transferred to the WDXRF cup and holder as shown in Figure S2c and d. The XRF measurement (Bruker, S8 Tiger model) was carried out using Rh X-ray tubes (40 kV and 10 mA) under AtmHe atmosphere. Table S1 shows that the culture medium with 500 ppm Cu-BTC exhibits 491 ppm. Whilst, other samples show no copper.


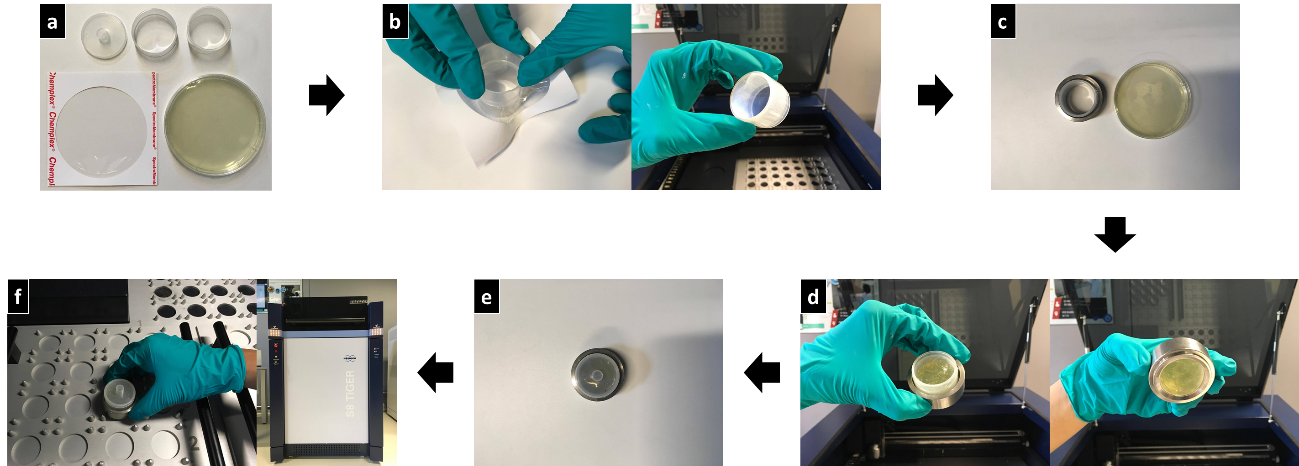


**Figure S1.** Flowchart of XRF sample preparation (for culture medium with Cu-BTC).

**
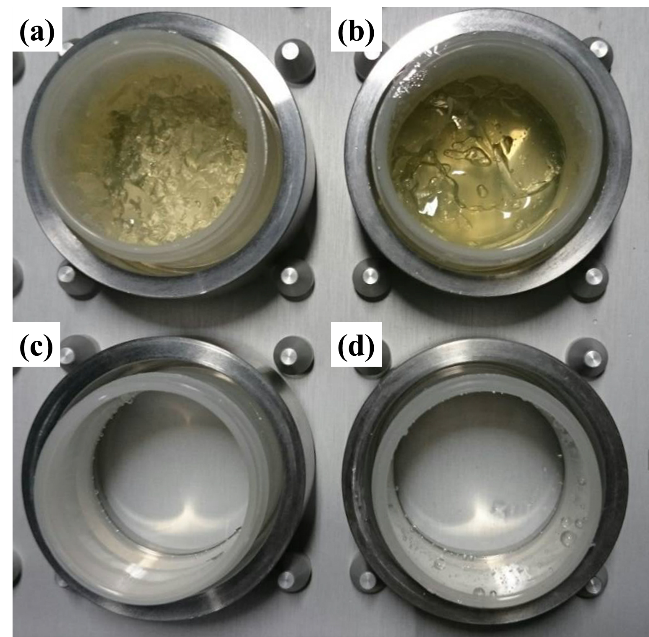
**

**Figure S2.** Sample preparation of XRF measurment (a) Culture medium, (b) Culture medium with Cu, (c) Filtered water from culture medium, and (d) Filtered water from culture medium with Cu-BTC.

**Table S1.** Cu concentration in samples measuring by XRF technique.

| **Samples** | **Cu found (ppm)** |
| --- | --- |
| Culture medium | 0 |
| Culture medium with 500 ppm Cu-BTC | 491.0 |
| The filtered water from the culture medium without Cu-BTU | 0 |
| The filtered water from the culture medium with 500 ppm Cu-BTC | 0 |
